# Lafora Disease: Report of a Rare Entity

**DOI:** 10.7759/cureus.6793

**Published:** 2020-01-28

**Authors:** Younis Al Mufargi, Asim Qureshi, Abdullah Al Asmi

**Affiliations:** 1 Neurology/Medicine, Sultan Qaboos University, Muscat, OMN; 2 Histopathology, Kings Mill Hospital, Nottingham, GBR; 3 Neurology, Sultan Qaboos University, Muscat, OMN

**Keywords:** lafora disease, skin biopsy, neurodenerative disease

## Abstract

Lafora disease is a rare, genetic, glycogen metabolism disorder inherited as autosomal recessive characterized by the presence of inclusion bodies, known as Lafora bodies, within the cytoplasm of the cells in the heart, liver, muscle, and skin. Lafora disease presents as a neurodegenerative disorder that causes impairment in the development of cerebral cortical neurons. We present here a case of Lafora disease that presented with progressive myoclonus epilepsy (PME) and investigated at our center. She was diagnosed to have Lafora disease with typical histological findings on skin biopsy and was found to be positive for the pathogenic mutation on genetic testing.

## Introduction

Lafora disease is rare worldwide; however, has a higher incidence among children and adolescents of positive ancestry. The clinical features of Lafora disease begin in the early adolescent years and are progressive. In few cases, learning difficulty may be observed as early as five years of age. Lafora disease presents as a neurodegenerative disorder that causes impairment in the development of cerebral cortical neurons. It is rare, progressive, fatal myoclonic epilepsy transmitted in an autosomal recessive pattern due to mutation of the EPM2A gene encoding laforin or NHLRC1/EPM2B [1]. The absence of either protein results in poorly branched, hyper phosphorylated glycogen, which precipitates, aggregates, and accumulates into Lafora bodies. Evidence from Lafora disease, genetic mouse models indicates that these intracellular inclusions are a principal driver of neurodegeneration and neurological disease. Laforin recruits malin to parts of glycogen molecules where overlying long glucose chains are forming, so as to counteract further chain extension [2]. In the absence of either laforin or malin function, long glucose chains in specific glycogen molecules extrude water, form double helices and drive precipitation of those molecules, which over time accumulate into Lafora bodies. It is characterized clinically by the triad of seizures, myoclonus, and dementia [3]. The onset of clinical manifestation usually starts in the range of 8-19 years of age and peaks around 15 years of age [4].

## Case presentation

A 15-year-old girl presented to ED of a tertiary care hospital with a history of frequent seizures and cognitive dysfunction. Her symptoms started at 13 years of age with occasional uncontrollable jerky movements of upper extremities followed later by generalized tonic-clonic seizures. The patient was started on anti-epileptics including sodium valproate, levetiracetam, and clonazepam with reasonable seizure control. However, later she started to have breakthrough seizures despite compliance with treatment. The family also noted a decline in her cognitive function in the form of difficulty doing schoolwork and decline in memory. She also experienced unsteady gait. There was no relevant family history, although the parents were first cousins.

On clinical examination, she was alert, conscious, and cooperative. She did not have any muco-cutaneous manifestations. Examination of the central nervous system revealed normal cranial nerves, motor, and sensory systems. There was cognitive dysfunction, and she was mildly disoriented. Myoclonic jerks were noted. She had bilateral mild cerebellar signs and ataxic gait. 

Her routine blood test blood tests were unremarkable. Serum B12, folate, and copper were within normal range. On electroencephalogram (EEG) there were continuous generalized spikes in a background of diffuse slowing in the range of theta and delta waves discharges over both hemispheres.

The patient was started on anti-epileptic medications which were built up in order to control her seizures which were achieved with reasonable seizure control. However, later she started to have breakthrough seizures despite compliance with treatment. She was taking sodium valproate, levetiracetam, and clonazepam.

**Figure 1:**
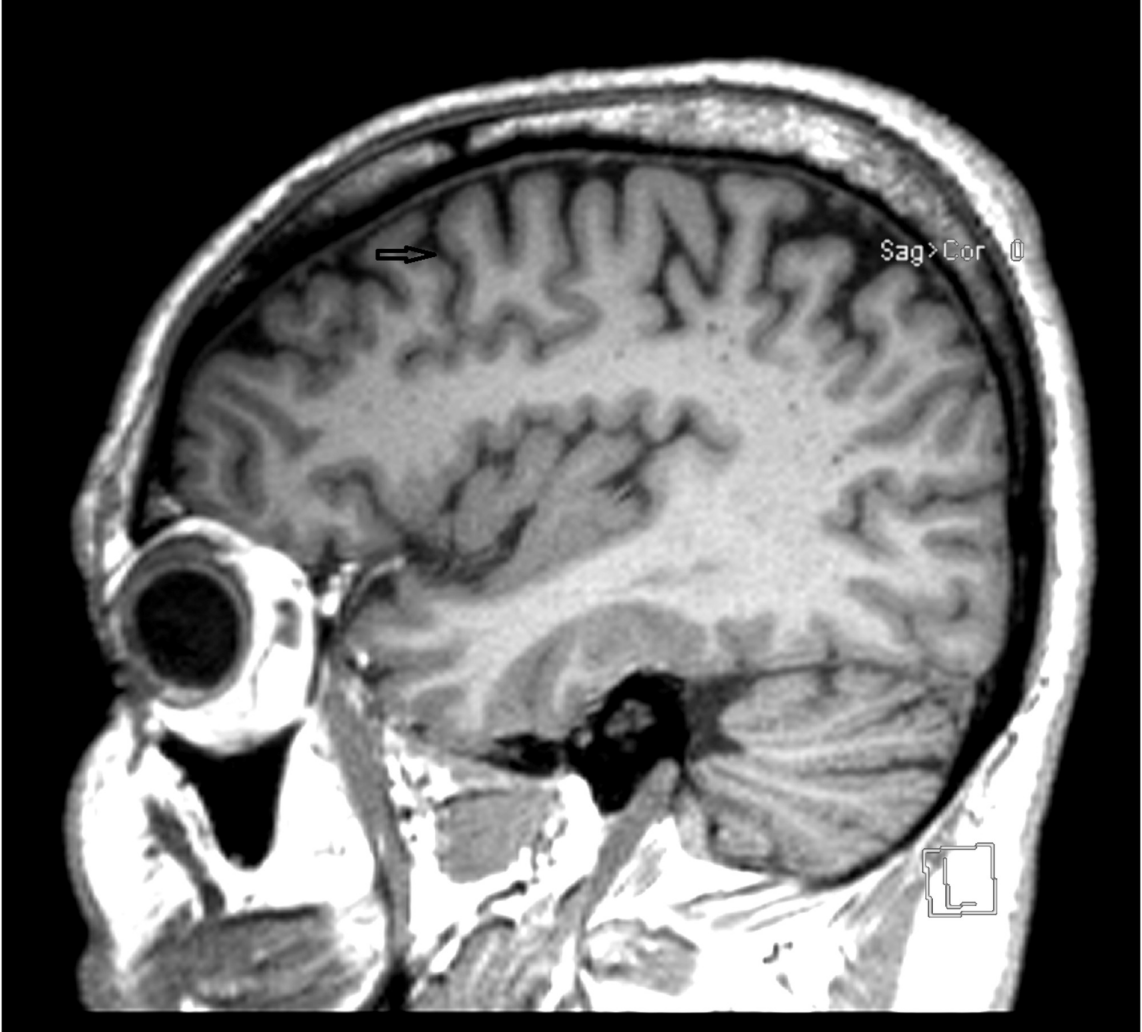
MRI of the brain.

**Figure 2:**
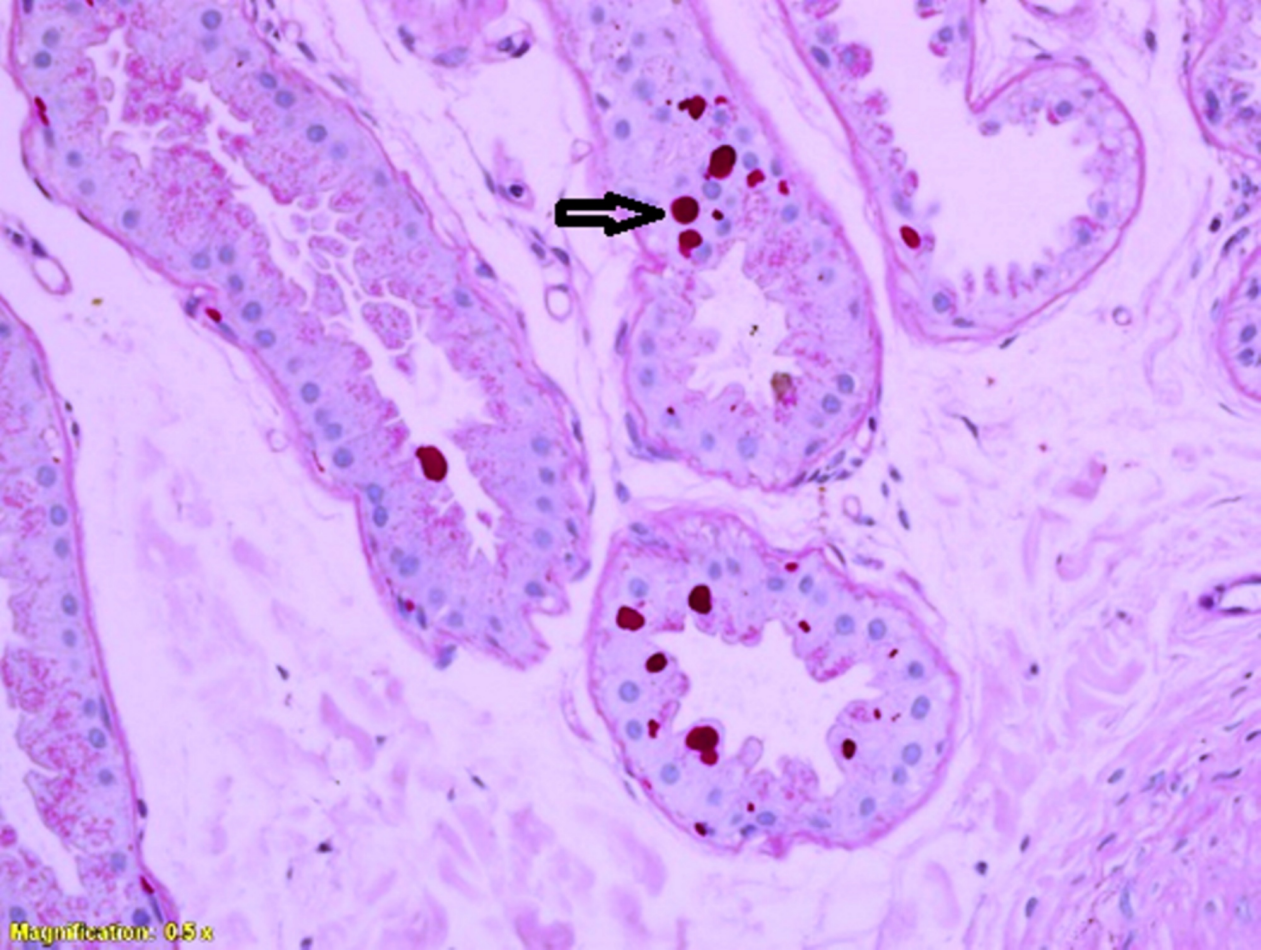
PAS stained slide showing Lafora bodies in the glandular epithelium. PAS, periodic acid-Schiff

The cranial MRI showed mild cerebellar and cerebral atrophy (Figure 1). Axillary skin biopsy revealed intra-cytoplasmic inclusion bodies in apocrine glands (Figure 2). These were positive by periodic acid-Schiff (PAS) at 40× magnification.

 

 

**Figure 3:**
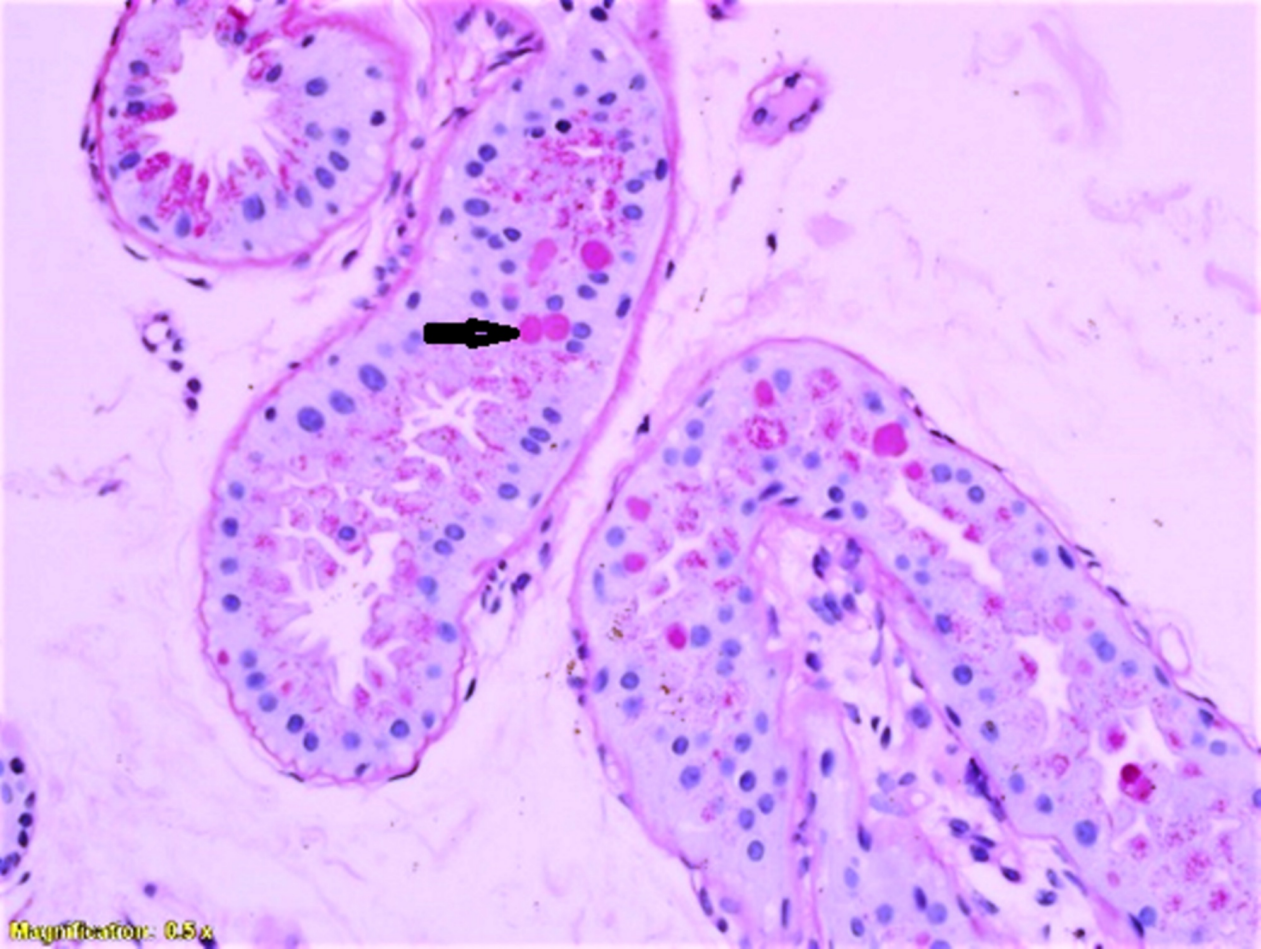
PAS with diastase showing washout of stain from the Lafora bodies in the glandular epithelium. PAS, periodic acid-Schiff

These inclusions demonstrated diastase resistance as shown in Figure 3 (at 40× magnification). Histological findings were consistent with a diagnosis of Lafora bodies. The genetic testing also confirmed the diagnosis of Lafora disease with a positive mutation on the NHLRC1 gene.

## Discussion

Symptoms of Lafora disease begin to develop during the early adolescent years, and symptoms progress as time passes. In the years before then, there is generally no indication of the presence of the disease, though in a few cases, the disease presents as a learning disorder around five years of age. The disease usually manifests in previously healthy adolescents, and death commonly occurs within 10 years of symptom onset. Lafora disease is caused by loss-of-function mutations in EPM2A or NHLRC1, which encode laforin and malin, respectively [2]. A Spanish neuropathologist called Gonzalo Rodríguez Lafora first described Lafora disease in an adolescent patient suffering from fatal, progressive myoclonic epilepsy [3-4]. Other common signs and symptoms associated with Lafora disease are behavioral changes due to the frequency of seizures. Over time those affected with Lafora disease have brain changes that cause confusion, speech difficulties, depression, decline in intellectual function, impaired judgement, and impaired memory. If areas of the cerebellum are affected by seizures, it is common to see problems with speech, coordination, and balance in Lafora patients. He described “intracellular amyloid bodies” in the brain and spinal cord of that patient. A characteristic Lafora body is periodic acid-Schiff (PAS)-positive diastase-resistant inclusions commonly seen in the gray matter of the brain. These bodies are free-lying and located mainly in the large pyramidal cells of the third and fifth layers of the cerebral cortex and mainly in the perikaryon [5-6]. Lafora bodies can be seen in the myoepithelial cells of the secretory acini of the apocrine sweat glands and the eccrine and apocrine sweat duct cells [7]. Skin biopsy from the axilla is preferable and often diagnostic because of the higher number of the sweat glands whose PAS-positive inclusion bodies can be more easily detected [8]. Fourteen patients from five tribes in Oman were found to have the same mutation, EPM2B-c.468-469delAG. In an earlier published series Turnball et al. describes 14 patients. It is noteworthy that the disease onset and death were the same; 14 years at onset and 21 years at death [9].

As reported in other parts of the world [10-11], our case also showed typical histopathological and genetic findings consistent with Lafora disease.

## Conclusions

Lafora disease should be considered in the differential diagnosis of patients who present with progressive myoclonus epilepsy (PME) especially with early cognitive decline in appropriate geographic setting with a high frequency of first degree marriage. We present here a case of Lafora disease that presented with PME and investigated at our center. She was diagnosed to have Lafora disease with typical histological findings on skin biopsy and was found to be positive for the pathogenic mutation on genetic testing.
